# Apgar Score Plus Umbilical Artery pH and Adverse Neonatal Outcomes in Very Preterm Infants

**DOI:** 10.1001/jamanetworkopen.2025.57913

**Published:** 2026-02-06

**Authors:** Harald Ehrhardt, Soodabeh Behboodi, Rolf F. Maier, Adrien M. Aubert, Ulrika Ådén, Birte Staude, Elizabeth S. Draper, Anna Gudmundsdottir, Veronica Siljehav, Heili Varendi, Tom Weber, Michael Zemlin, Jennifer Zeitlin

**Affiliations:** 1 Division of Neonatology and Pediatric Intensive Care Medicine, Department of Pediatrics and Adolescent Medicine, University Medical Center Ulm, Ulm, Germany; 2 Now with Department of Neonatology, Charité–Universitätsmedizin Berlin, Berlin, Germany; 3 Centre for Research in Epidemiology and Statistics, Obstetrical, Perinatal and Pediatric Lifecourse Epidemiology, Université Paris Cité, Institut National de la Santé et de la Recherche Médicale, Institut National de la Recherche pour l’Agriculture, l’Alimentation et l’Environnement (INRAE), Paris, France; 4 Children’s Hospital, University Hospital, Philipps University Marburg, Marburg, Germany; 5 Department of Women’s and Children’s Health, Karolinska Institutet, Stockholm, Sweden; 6 Department of Bioclinical Sciences, Linköping University, Linköping, Sweden; 7 Department of Health Sciences, University of Leicester, Leicester, United Kingdom; 8 University of Tartu, Tartu University Hospital, Tartu, Estonia; 9 University of Copenhagen, Copenhagen, Denmark; 10 Hospital for General Pediatrics and Neonatology, Saarland University Medical Center, Homburg, Germany

## Abstract

**Importance:**

The Apgar score, the first clinical assessment to direct measures to stabilize newborn infants, is also used for risk assessment. Its accuracy in estimating outcomes remains poor among very preterm (VPT) infants.

**Objective:**

To assess the utility of the combined 5-minute Apgar score and umbilical artery pH (UA-pH) for estimating risks of mortality and severe neonatal morbidity among VPT infants.

**Design, Setting, and Participants:**

This cohort study (Effective Perinatal Intensive Care in Europe [EPICE]) analyzed infants born at less than 32 weeks’ gestation between April 2011 and September 2012 across 11 European countries. All liveborn VPT infants with Apgar scores and UA-pH data were included. Data were analyzed between February and December 2025.

**Exposures:**

Apgar score at 5 minutes and UA-pH. The Apgar score was classified as lower than 7 and 7 or higher, and the UA-pH values were categorized as low (<7.20) and normal (≥7.20). Four groups that combined these 2 measures were defined: Apgar score lower than 7 and low UA-pH; Apgar score lower than 7 and normal UA-pH; Apgar score 7 or higher and low UA-pH; and Apgar score 7 or higher and normal UA-pH.

**Main Outcomes and Measures:**

Combined outcome of mortality and/or any adverse morbidity (intraventricular hemorrhage [IVH] >grade 2, cystic periventricular leukomalacia, moderate or severe bronchopulmonary dysplasia [BPD], retinopathy of prematurity ≥stage 2, and necrotizing enterocolitis). Modified Poisson regression was used to estimate relative risks (RRs) between the exposure and the combined mortality and morbidity outcome and 3 individual components: mortality, IVH, and BPD. Models were adjusted for perinatal variables associated with Apgar score and UA-pH and adverse neonatal outcomes.

**Results:**

Of 7900 liveborn infants in the EPICE cohort, 4174 (52.8%) had information on Apgar score and UA-pH. These infants included 2249 males (53.9%) and had a median [IQR] gestational age of 29.9 [27.9-31.0] weeks and median [IQR] birth weight of 1240 [960-1520] g. A total of 367 infants (8.8%) had an Apgar score 7 or higher but a low UA-pH, 558 (13.4%) had an Apgar score lower than 7 but a normal UA-pH, and 196 (4.7%) had an Apgar score lower than 7 and a low UA-pH. Infants with an Apgar score lower than 7 had a higher frequency of the combined outcome among those with a normal UA-pH (270 [48.4%] vs 596 [19.5%]) and a low UA-pH (108 [55.1%] vs 596 [19.5%]), with similar adjusted RRs (ARRs; low: 1.4 [95% CI, 1.2-1.7]; normal: 1.4 [95% CI, 1.3-1.6]). For mortality risk, associations were robust for an Apgar score lower than 7 and a low UA-pH (ARR, 2.4; 95% CI, 1.7-3.3) and absent with an Apgar score of 7 or higher and a low UA-pH (ARR, 1.2; 95% CI, 0.8-1.8). IVH risk was increased in all 3 subcategories, including an Apgar score of 7 or higher with a low UA-pH (ARR, 2.0; 95% CI, 1.3-3.0). BPD risk was associated only with an Apgar score lower than 7 and a normal UA-pH (ARR, 1.4; 95% CI, 1.2-1.7).

**Conclusions and Relevance:**

In this cohort study of VPT infants, combining information on UA-pH with the 5-minute Apgar score was associated with improved accuracy in estimating the risk of some adverse outcomes—notably mortality and IVH, which occurred soon after birth. These results highlight the importance of exploring the associations of early markers of risk with neonatal mortality and key neonatal morbidities separately.

## Key Points

**Question:**

Is the addition of umbilical artery pH (UA-pH) to the 5-minute Apgar score associated with more accurate risk estimates for adverse neonatal outcome?

**Findings:**

In this cohort study of 4174 liveborn VPT infants, those with Apgar score lower than 7 had similar risks of a composite mortality and morbidity outcome regardless of low or normal UA-pH. Mortality risk was highest for infants with an Apgar score lower than 7 and a low UA-pH, severe intraventricular hemorrhage risk was elevated when either Apgar score or UA-pH was low, and only Apgar score lower than 7 and a normal UA-pH was associated with bronchopulmonary dysplasia risk.

**Meaning:**

The findings of this study suggest that combining information on UA-pH with Apgar score is associated with improved estimation of risk for individual components of composite neonatal outcomes.

## Teaser

This cohort study evaluates whether combining measures of umbilical artery pH with 5-minute Apgar scores could improve neonatal mortality and morbidity risk assessment among infants born at less than 32 weeks’ gestation.

## Introduction

Very preterm (VPT) birth at less than 32 weeks’ gestation can lead to severe neonatal morbidities, including intraventricular hemorrhage (IVH) and bronchopulmonary dysplasia (BPD), which are related to long-term health difficulties, neurodevelopmental impairment, and reduced quality of life.^1,2,3,4,5^ An unmet challenge for clinicians and researchers is to estimate infants’ risk for developing these morbidities to target preventive care. Patient characteristics and perinatal management, including gestational age (GA), birth weight, sex, fetal .growth restriction, infection, delivery in a level III hospital, and receipt of antenatal corticosteroids (ACS), constitute established variables in neonatal morbidity. However, the accurate estimation of both short and long-term outcomes based on clinical items or biomarkers before or soon after birth remains an unmet need.^6,7,8,9,10,11^

The Apgar score evaluated at 1, 5, and 10 minutes of life is the first clinical assessment after birth that directs stabilization measures in the delivery room. For term infants, a reliable association between low 5-minute Apgar scores and adverse neonatal outcomes has been established in large register cohorts. This measure is thus increasingly used to assess risk, despite warnings from its inventor and organizations, such as the American College of Obstetricians and Gynecologists, about its use to specify individual risks, in large part because of poor validity and reproducibility of Apgar scoring.^12,13,14,15,16,17,18^

The accuracy of the 5-minute Apgar score for estimating in-hospital mortality decreases with declining GA. One reason for this decrease may be uncertainty about how to score the individual items in preterm infants, raising questions about the measure’s utility in the VPT population.^19,20^ No association was detected between the 5-minute Apgar score and severe brain injury—defined as IVH higher than grade 2 or periventricular leukomalacia within the iNeo (International Network for Evaluating Outcomes of Neonates) research collaborative—in preterm infants born between 24 and 28 weeks’ GA.^21^ A recent analysis of the Effective Perinatal Intensive Care in Europe (EPICE) cohort showed more consistent associations of the Apgar score with adverse neonatal outcomes, but country-specific factors in 5-minute Apgar scoring affected international comparative analyses.^22^ The umbilical artery pH (UA-pH), which reflects the deficit in fetal oxygen supply within the period immediately before birth, is also associated with clinical outcomes.^23^ A low UA-pH demonstrates a much better association with hypoxic-ischemic encephalopathy in infants at term.^24,25^

Whether adding information on the UA-pH to the Apgar score is associated with improved risk estimation for adverse neonatal outcomes among VPT infants has not been investigated, to our knowledge. To address this gap in the literature, we used data from EPICE, a population-based multinational European cohort of VPT births to assess the utility of the combined 5-minute Apgar score and UA-pH for estimating risks of mortality and severe neonatal morbidity.

## Methods

### Design, Data Source, and Population

The EPICE cohort included all stillbirths and live births from 22 to 31 weeks’ gestation, in 19 regionally and organizationally diverse regions in 11 European countries over a 12-month period (6 months in French regions) between April 2011 and September 2012.^26^ Data were collected from obstetrical and neonatal records using a pretested standardized instrument. The analysis sample contained all live births in the EPICE cohort with data on the 5-minute Apgar score and UA-pH. Ethics approval was obtained from all regional and/or hospital ethics committees or regional ethical review boards as required by law and was additionally approved by the French Advisory Committee on Use of Health Data in Medical Research and the French National Commission for Data Protection and Liberties. Active or passive informed parental consent was obtained as required by national legislation of participating countries. We followed the Strengthening the Reporting of Observational Studies in Epidemiology (STROBE) reporting guideline.

### Exposures, Outcomes, and Perinatal Variables

#### Exposures

The exposures studied were the 5-minute Apgar score and the UA-pH. We used the 5-minute Apgar score for its superior predictive value compared with the 1-minute assessment after birth and to allow comparability to other large studies on the topic.^12,19,21,27,28^ An Apgar score cutoff of 7 was chosen in conformity with previous studies on the topic in which scores lower than 7 were associated with adverse outcomes.^12,19,21,22^ We classified the Apgar score into lower than 7 and 7 or higher and the UA-pH values into low (<7.20) and normal (≥7.20) and created a combined variable with 4 categories (Apgar score <7 and UA-pH <7.20; Apgar score <7 and UA-pH ≥7.20; Apgar score ≥7 and UA-pH <7.20; and Apgar score ≥7 and UA-pH ≥7.20). These categories were selected because they have been used in the literature and enabled us to have sufficient numbers of cases in each group for robust analyses.^23,29,30,31^

#### Outcomes

To take into consideration potential trade-offs between mortality and adverse morbidity, our main outcome was the combined outcome of mortality and/or any adverse morbidity (IVH >grade 2, cystic periventricular leukomalacia, necrotizing enterocolitis requiring surgery or peritoneal drainage, retinopathy of prematurity >stage 2, and moderate or severe BPD). We also analyzed 3 select components of this composite: mortality, IVH, and BPD. The prevalence rates of retinopathy of prematurity (2.8%) and necrotizing enterocolitis (2.0%) were too low to allow a reliable analysis with our 4-category exposure variable. Mortality was assessed on all live births, IVH on all infants with available cranial ultrasonography data, and BPD on infants surviving to 36 weeks’ postmenstrual age.

#### Perinatal Variables

We selected perinatal variables that may be independently associated with Apgar score and UA-pH and the study outcomes. These variables included maternal age; parity; multiple pregnancy; antepartum hemorrhage after 20 weeks’ GA; admission for preterm labor or contractions after 20 weeks; preeclampsia, eclampsia, or HELLP (hemolysis, elevated liver enzymes, and low platelets) syndrome or other indication; and mode of delivery. GA was the obstetrical estimate based on information from the last menstrual cycle and routine ultrasonography measures. Preterm premature rupture of membranes (PPROM) was recorded when the onset was more than 12 hours before delivery. Any administration of ACS was considered regardless of the number of doses or timing, and inborn status was defined as birth in the neonatal unit where the infant was hospitalized for the first consecutive 48 hours. For the infant, we included information on sex and birth weight for GA. Small for gestational age (SGA) was classified as birth weight lower than the third percentile, in the third to ninth percentile, or in the 10th or higher percentile, based on growth references developed for the cohort.^32^

#### Missing Data

Given that data were abstracted from medical records, we were able to retrieve measures of 5-minute Apgar score and UA-pH only when they were recorded in notes. Often, UA-pH was missing because of early labor ward death or because of differences in unit practices within countries. Overall, data on 5-minute Apgar score and UA-pH were available for more than 50% of the total live births. Within the sample, missing data were low (<2%) for the perinatal and outcome variables.

### Statistical Analysis

Data were analyzed between February and December 2025. We compared characteristics of cases with missing Apgar score and UA-pH and cases with available data to assess for potential biases. We then compared differences in the distribution of perinatal characteristics and management by Apgar score and UA-pH categories using χ^2^ tests. After describing the main outcomes by Apgar score and UA-pH group, we estimated unadjusted and adjusted relative risks (ARRs) with modified Poisson regression models.^33^ One model included only country with no other covariables, and a second model was adjusted for country, maternal age, perinatal variables (GA, SGA, sex, multiple birth, and congenital anomaly), pregnancy complications (preterm rupture of membranes and hypertensive disorders of pregnancy), parity, mode of delivery, ACS use, and inborn status. We performed a complete case analysis because of the difficulty of defining mechanisms for the missing UA-pH data given the association with varying unit practices in participating countries.

We carried out 4 sensitivity analyses by running our unadjusted and final adjusted models (1) after removing countries with a high proportion of missing UA-pH data; (2) using varying adjustment strategies to assess the models’ sensitivity to the choice of covariables; (3) taking into consideration clustering within units; and (4) varying the cutoff for UA-pH by using 7.10 instead of 7.20.

*P* < .05 was considered statistically significant using 2-sided tests. Analyses were conducted using Stata, version 15.0 (StataCorp LLC).

## Results

### Cohort Characteristics

**Figure 1.**
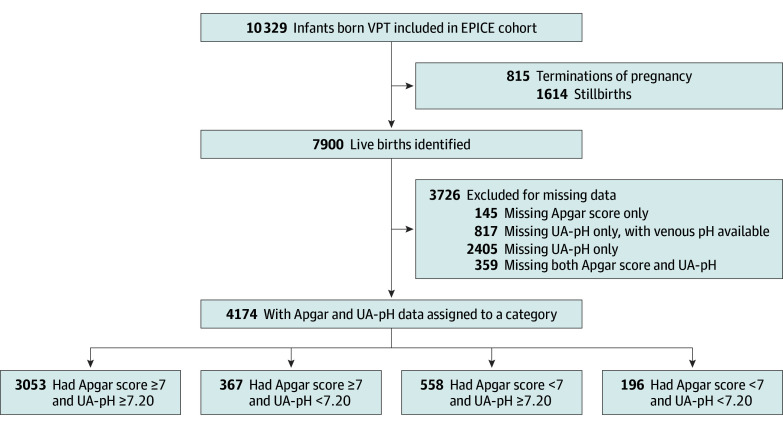
Flowchart of Patient Allocation to the Apgar Score and Umbilical Artery pH (UA-pH) Categories EPICE indicates Effective Perinatal Intensive Care in Europe; VPT, very preterm.

Among the 10 329 total births included in the EPICE cohort, 7900 (76.5%) were live births, of whom 4174 (52.8%) had data on both the 5-minute Apgar score and UA-pH (Figure 1). The 5-minute Apgar score was missing for 504 infants (6.4%), whereas UA-pH was missing for 3581 infants (45.3%). Missing UA-pH was either because the value was not recorded or venous UA-pH was reported instead.

In the analysis sample of 4174 infants, 2249 (53.9%) were male and 1925 (46.1%) were female, with a median [IQR] GA of 29.9 [27.9-31.0] weeks and a median [IQR] birth weight of 1240 [960-1520] g (Table 1). A total of 754 infants (18.1%) had an Apgar score lower than 7, while 563 (13.5%) fulfilled the criterion of low UA-pH (<7.20). When these variables were combined, 367 infants (8.8%) had an Apgar score of 7 or higher but a low UA-pH, 558 (13.4%) had an Apgar score lower than 7 but a normal UA-pH (≥7.20), and 196 (4.7%) had an Apgar score lower than 7 and a low UA-pH.

**Table 1.** Maternal and Neonatal Characteristics of Very Preterm Liveborn Infants With Less Than 32 Weeks’ Gestation, Stratified by Apgar Score and Umbilical Artery pH Category

Characteristic	No. (%)^a^					*P* value^b^
	All births	Apgar score ≥7		Apgar score <7		
		and UA-pH ≥7.20	and UA-pH <7.20	and UA-pH ≥7.20	and UA-pH <7.20	
Maternal age, y						
<25	678 (16.3)	463 (15.2)	73 (20.1)	103 (18.5)	39 (20.1)	.053
25-34	2466 (59.3)	1845 (60.6)	202 (55.5)	311 (55.8)	108 (55.7)	
≥35	1018 (24.5)	739 (24.3)	89 (24.5)	143 (25.7)	47 (24.2)	
Missing data, No.	12	2	1	3	6	
Parity						
Primiparous	2335 (56.5)	1725 (57.0)	208 (58.3)	299 (54.1)	103 (53.1)	.39
Multiparous	1795 (43.5)	1301 (43.0)	149 (41.7)	254 (45.9)	91 (46.9)	
Missing data, No.	44	2	5	10	27	
Type of pregnancy						
Singleton	2928 (70.2)	2061 (67.5)	279 (76.2)	420 (75.3)	168 (85.7)	<.001
Multiple	1245 (29.8)	992 (32.5)	87 (23.8)	138 (24.7)	28 (14.3)	
Missing data, No.	1	0	0	1	0	
Preeclampsia, eclampsia, or HELLP						
No	3379 (82.6)	2471 (82.5)	276 (75.8)	475 (86.4)	157 (86.7)	<.001
Yes	711 (17.4)	524 (17.5)	88 (24.2)	75 (13.6)	24 (13.3)	
Missing data, No.	84	15	8	3	58	
PPROM: >12 h						
No	3034 (74.1)	2207 (73.6)	299 (82.4)	392 (71.3)	136 (74.7)	.001
Yes	1060 (25.9)	792 (26.4)	64 (17.6)	158 (28.7)	46 (25.3)	
Missing data, No.	80	14	8	4	54	
Any ACS use						
No	388 (9.4)	232 (7.7)	44 (12.1)	79 (14.3)	33 (17.1)	<.001
Yes	3747 (90.6)	2794 (92.3)	321 (87.9)	472 (85.7)	160 (82.9)	
Missing data, No.	39	3	7	2	27	
Inborn						
No	389 (9.4)	242 (7.9)	51 (13.9)	65 (12.1)	31 (16.3)	<.001
Yes	3749 (90.6)	2804 (92.1)	315 (86.1)	471 (87.9)	159 (83.7)	
Missing data, No.	36	6	22	1	7	
Mode of delivery						
Vaginal	1160 (28.1)	837 (27.7)	98 (26.8)	165 (29.9)	60 (30.8)	<.001
Vaginal instrumental	102 (2.5)	67 (2.2)	14 (3.8)	13 (2.4)	8 (4.1)	
Prelabor cesarean	1774 (42.9)	1286 (42.6)	190 (51.9)	214 (38.8)	84 (43.1)	
Intrapartum cesarean	1098 (26.6)	832 (27.5)	64 (17.5)	159 (28.9)	43 (22.1)	
Missing data, No.	40	1	7	1	31	
Neonatal sex						
Male	2249 (53.9)	1623 (53.2)	211 (57.5)	302 (54.1)	113 (57.7)	.30
Female	1925 (46.1)	1430 (46.8)	156 (42.5)	256 (45.9)	83 (42.3)	
Gestational age, completed wk						
≤24	221 (5.3)	92 (3.0)	19 (5.2)	75 (13.4)	35 (17.9)	<.001
25	207 (5.0)	116 (3.8)	11 (3.0)	63 (11.3)	17 (8.7)	
26	278 (6.7)	185 (6.1)	19 (5.2)	57 (10.2)	17 (8.7)	
27	377 (9.0)	258 (8.5)	26 (7.1)	74 (13.3)	19 (9.7)	
28	509 (12.2)	363 (11.9)	45 (12.3)	79 (14.2)	22 (11.2)	
29	600 (14.4)	452 (14.8)	57 (15.5)	66 (11.8)	25 (12.8)	
30	876 (21.0)	694 (22.7)	88 (24.0)	69 (12.4)	25 (12.8)	
31	1106 (26.5)	893 (29.2)	102 (27.8)	75 (13.4)	36 (18.4)	
SGA, centiles, intrauterine charts						
<3	877 (21.0)	620 (20.3)	125 (34.1)	87 (15.6)	45 (23.0)	<.001
3-9	480 (11.5)	350 (11.5)	47 (12.8)	62 (11.1)	21 (10.7)	
≥10	2817 (67.5)	2083 (68.2)	195 (53.1)	409 (73.3)	130 (66.3)	
Severe congenital malformation						
No	3780 (90.6)	2782 (91.1)	345 (94.0)	481 (86.2)	172 (87.8)	<.001
Yes	394 (9.4)	271 (8.9)	22 (6.0)	77 (13.8)	24 (12.2)	

Abbreviations: ACS, antenatal corticosteroids; HELLP, hemolysis, elevated liver enzymes, and low platelets; NA, not available; PPROM, preterm premature rupture of membranes; SGA, small for gestational age; UA-pH, umbilical artery pH.

^a^ Total number of patients differs because of missing values for individual covariables due to death before diagnosis or missing data.

^b^ *P* values were calculated using χ^2^ tests.

When examining differences between infants with and without data on 5-minute Apgar score and UA-pH (eTables 1 and 2 in Supplement 1), country differences in measurement were observed, with higher proportions of infants (>75%) with data in Poland, Estonia and Germany and lower proportions of infants (<35%) with data in Italy and Portugal. After adjustment for country, the characteristics associated with missing Apgar score and UA-pH data were multiple birth (1216 [32.6%]); birth at less than 26 weeks’ gestation (737 [19.8%]); vaginal delivery (1334 [36.4%]); outborn status (471 [13.5%]); pregnancy complicated by preeclampsia, eclampsia, or HELLP (442 [12.2%]); and no ACS use (667 [18.1%]).

Additional patient baseline characteristics by 5-minute Apgar score and UA-pH categories are detailed in Table 1. Infants with an Apgar score lower than 7 and a low UA-pH were more likely to be from singleton pregnancies and to belong to the lowest GA stratum (≤24 weeks). Preeclampsia, eclampsia, or HELLP was less frequently observed in infants with an Apgar score lower than 7 regardless of the UA-pH, and the rate of ACS use was lower. Meanwhile, cases with an Apgar score of 7 or higher and a low UA-pH had the lowest frequencies of PPROM and congenital anomalies and the highest rates of prelabor cesarean delivery and SGA (<3 percentile). Infants in the Apgar score of 7 or higher and normal UA-pH category had the highest likelihood of being of multiple births and having received ACS and the lowest rate of being born in the lowest GA stratum.

### Association Between 5-Minute Apgar Score and UA-pH Subcategories and Adverse Neonatal Outcomes

**Figure 2.**
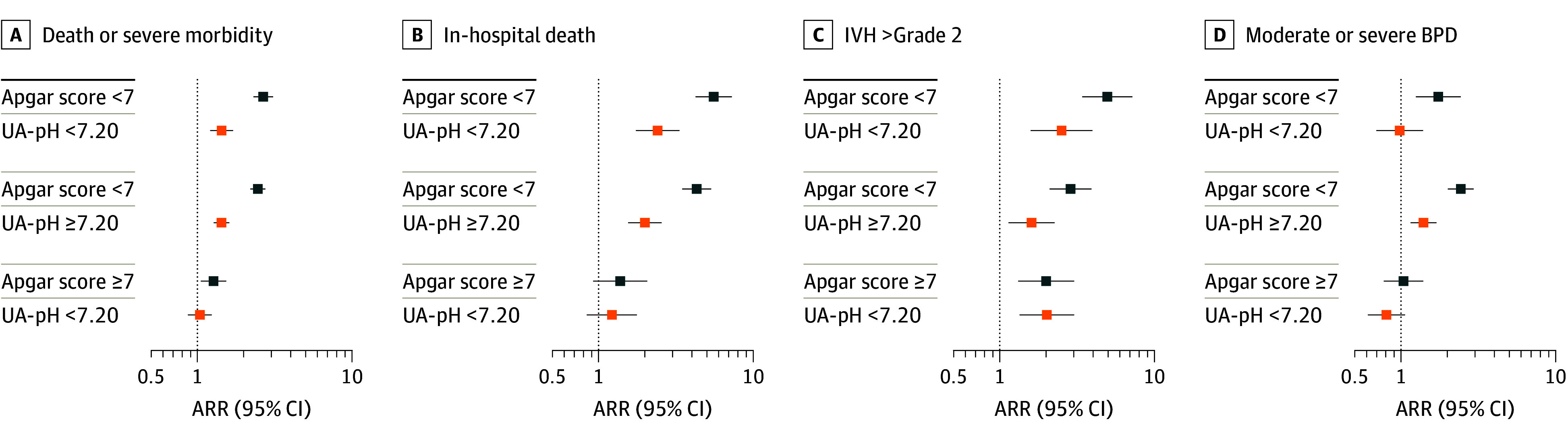
Dot Graph of Severe Acute Outcomes in Infants With Less Than 32 Weeks’ Gestation by Apgar Score and Umbilical Artery pH (UA-pH) Data were analyzed using modified Poisson regression without adjustments except for country (blue squares) and after adjustment in full model (orange squares) for maternal age, perinatal variables (gestational age, small for gestational age, sex, multiple birth, congenital anomaly), pregnancy complications (preterm rupture of membranes, hypertensive disorders of pregnancy), parity, mode of delivery, antenatal steroid administration, and inborn status. Infants with Apgar score of 7 or higher and UA-pH of 7.20 or higher served as the reference group in the modified Poisson regression models. ARR indicates adjusted relative risk; IVH, intraventricular hemorrhage; BPD, bronchopulmonary dysplasia.

The group at highest risk of mortality and/or adverse morbidity had both an Apgar score lower than 7 and a low UA-pH (108 [55.1%]), followed by infants with an Apgar score lower than 7 and a normal UA-pH (270 [48.4%]) (Table 2). Infants with an Apgar score of 7 or higher and a low UA-pH had somewhat higher proportions of the primary outcome than those with an Apgar score of 7 or higher and a normal UA-pH (94 [25.6%] vs 596 [19.5%]). After adjustment for country of birth and perinatal factors, there was no difference in the composite mortality and morbidity outcome associated with an Apgar score lower than 7 and low or a normal UA-pH (low: ARR, 1.4 [95% CI, 1.2-1.7]; normal: ARR, 1.4 [95% CI, 1.3-1.6]) (Figure 2; coefficients are presented in eTable 3 in Supplement 1). The association was high in magnitude between mortality risk and an Apgar score lower than 7 and a low UA-pH (ARR, 2.4; 95% CI, 1.7-3.3) (Figure 2; coefficients are presented in eTable 3 in Supplement 1) compared with normal UA-pH (ARR, 2.0; 95% CI, 1.6-2.6). In contrast, cases with a low UA-pH and an Apgar score of 7 or higher had no increased risk for mortality compared with those infants with a normal UA-pH (ARR, 1.2; 95% CI, 0.8-1.8). For IVH risk, the association was robust in cases with an Apgar score lower than 7 and a low UA-pH (ARR, 2.5; 95% CI, 1.6-4.0) compared with normal UA-pH (ARR, 1.6; 95% CI, 1.1-2.3). An association was also found in infants with Apgar score of 7 or higher and a low UA-pH (ARR, 2.0; 95% CI, 1.3-3.0) compared with an Apgar score of 7 or higher and a normal UA-pH. In contrast, no associations were seen between most subcategories and BPD risk, except for an Apgar score lower than 7 and a normal UA-pH (ARR, 1.4; 95% CI, 1.2-1.7).

**Table 2.** Prevalence of Acute Adverse Neonatal Outcomes by Apgar Score and Umbilical Artery pH Category

Adverse neonatal outcomes	No. (%)^a^					*P* value^b^
	All births	Apgar score ≥7		Apgar score <7		
		With UA-pH ≥7.20	With UA-pH <7.20	With UA-pH ≥7.20	With UA-pH <7.20	
No.^a^	4174	3053	367	558	196	
Death or severe morbidity^c^	1068 (25.6)	596 (19.5)	94 (25.6)	270 (48.4)	108 (55.1)	<.001
In-hospital death	366 (8.8)	157 (5.1)	26 (7.1)	126 (22.6)	57 (29.1)	<.001
IVH >grade 2	222 (5.5)	107 (3.6)	26 (7.2)	56 (10.6)	33 (18.4)	<.001
BPD: moderate or severe	493 (13.0)	310 (10.8)	43 (12.6)	111 (25.6)	29 (20.6)	<.001

Abbreviations: BPD, bronchopulmonary dysplasia; IVH, intraventricular hemorrhage; UA-pH, umbilical artery pH.

^a^ Total number of patients differs because of missing values for individual covariables due to death before diagnosis or missing data: 103 for IVH (no cranial ultrasonography), 391 for BPD, 30 for necrotizing enterocolitis (NEC), and 104 for retinopathy of prematurity (ROP).

^b^ *P* values were calculated using χ^2^ tests.

^c^ The primary outcome of death or severe morbidity includes in-hospital death, IVH higher than grade 2, NEC, cystic periventricular leukomalacia, ROP higher than stage 2, and moderate or severe BPD.

### Sensitivity Analyses

Results of the unadjusted and adjusted final models were robust to removal of countries with high missing UA-pH data: greater than 65% after removing Italy (770 [67.9%]) and Portugal (624 [86.2%]) (eFigure 1 in Supplement 1) and greater than 50% after adding the UK (919 [52.7%]) and Sweden (139 [52.1%]) (eFigure 2 in Supplement 1). Similar results were found with alternative covariable specifications (eFigures 3, 4, and 5 in Supplement 1), when considering clustering within units (eFigure 6 in Supplement 1), and when using the cutoff of 7.10 for UA-pH (eFigure 7 in Supplement 1).

## Discussion

Our study illustrated the utility of combining information on UA-pH with 5-minute Apgar scores of VPT infants for estimating risks of death and/or key acute severe morbidities. While the risks of the composite outcome of mortality and morbidity did not differ among infants with an Apgar score lower than 7 and both low and normal UA-pH values, compared with infants with an Apgar score of 7 or higher and a normal UA-pH, the individual components of this composite showed differing associations. Infants with Apgar scores lower than 7 and a low UA-pH had the highest risk of death and IVH. Risk of IVH was also elevated for infants with an Apgar score higher than 7 and a low UA-pH. In contrast, BPD risk was only moderately elevated for the subgroup of infants with an Apgar score lower than 7 and a normal UA-pH. These results provide new knowledge relevant to clinical care and research and indicate directions for future research to improve the accuracy of current risk assessment tools by including these 2 easily applicable clinical and biomarker assessments.

Our results expand on previous studies on the association of the 5-minute Apgar score with adverse outcomes following VPT birth in the EPICE cohort.^22,34^ A recent study showed that an Apgar score lower than 7 was associated with in-hospital mortality and most adverse acute outcomes.^22^ Herein, we found that low UA-pH represents a relevant additional risk for early mortality and severe IVH. For outcomes occurring later in the neonatal course, such as BPD, the associations were less pronounced. It is well established that the longitudinal course in the neonatal intensive care unit and clinical management until the point of diagnosis at 36 weeks’ postmenstrual age have greater implications for BPD. Nonetheless, a question remains regarding the explanation for the increased risk for BPD in the group of infants with an Apgar score lower than 7 but with a normal UA-pH.

The initial analysis of the 5-minute Apgar score in the EPICE cohort did not detect an association between the Apgar score when categorized into 4 groups (SGA percentiles: ≤3, 4-6, 7-8, 9-10) and cognitive or motor outcomes at 5 years of age in the subcohort of 996 extremely preterm infants born at less than 28 weeks’ gestation.^34^ In contrast, in the total population of 7900 liveborn VPT infants born at less than 32 weeks’ gestation, an Apgar score lower than 7 was associated with most adverse acute outcomes, including mortality, IVH, and BPD.^22^ IVH and BPD are well known for their association with abnormal psychomotor development.^35,36^ Because of sample size limitations, our main analysis used UA-pH lower than 7.20. Although, reassuringly, sensitivity analyses with a lower cutoff of 7.10 yielded similar results, further subcategorization by the UA-pH value remains a research task for other larger cohorts. Future research should also assess the associations with neurodevelopmental outcomes.

It seems promising to test whether adding more clinical characteristics and/or routine laboratory values can improve the accuracy of risk assessment models. Our analyses combined with previous work within the multinational EPICE cohort underline the need to specify precise patient subcategories in accordance with current standards (eg, in oncology) to achieve optimal discrimination of risks and estimation of outcomes and to offer the therapy only to patients at risk and avoid exposing others unnecessarily. For optimal accuracy, it will be essential for future studies to focus on both short-term and long-term outcomes.

### Strengths and Limitations

The main strength of our study is the prospective design with data collection from 11 geographically and organizationally diverse countries across Europe. This design allowed analyses within a clinical setting and applicability of the results to diverse care settings. This and previous studies on the associations between Apgar score subcategories and the spectrum of adverse acute outcomes advance the knowledge on congruencies and disparities in associations. Furthermore, risk adjustment for country factors and baseline characteristics accounted for variations in care settings and management between the participating countries.^22^

Nevertheless, our study has limitations. First, UA-pH was not a standard clinical item throughout all countries; thus, there were large variations in data availability and often the data were missing (as can be expected), which might limit generalizability of the findings to these infants.^37^ However, sensitivity analyses that removed countries with high proportions of missing data gave similar results. We did not model the exposures as linear variables because, while there was agreement on lower cutoffs for the Apgar score, higher Apgar scores (ie, 8, 9, or 10) varied greatly across countries.^38^ We also had less knowledge about the linear distribution and impact of UA-pH and lacked the sample sizes for more complex modeling.

The data collection instrument did not include items such as diabetes during pregnancy or fetal anemia, which are risk factors for a low Apgar score and UA-pH, or postnatal stabilization measures such as initial respiratory support and fraction of inspired oxygen and the individual components of the Apgar score, limiting more precise risk estimates.^39^ Base excess data were not collected, which hampered the discrimination between metabolic acidosis and carbon dioxide transfusion from the mother, and appropriate cutoffs need to be defined. However, the literature on this topic does not show an additional advantage of estimating adverse outcomes in term infants when considering the negative base excess.^23,30,40^ Cohort data used in this study dates back from 2011 and 2012; however, there have not been major changes in outcomes since this period, and patterns of data across time from the National Center for Health Statistics also show that the frequency of the 5-minute Apgar score lower than 7 has not changed.^41^ Last, we classified the UA-pH threshold in accordance with the published literature on the topic, and our sensitivity analysis on cases with a UA-pH lower than 7.10 provided similar results.^23^

## Conclusions

Results of this cohort study indicate that, in VPT infants, the accuracy of the 5-minute Apgar score could be improved by combining it with the UA-pH. Future initiatives to develop risk assessment models should consider adding these items to test their added value. Such validation studies are needed before Apgar scores together with UA-pH and other items can be used to guide therapeutic decisions.^42,43,44^ These results further highlight the importance of exploring the associations of early markers of risk with neonatal mortality and key neonatal morbidities separately when developing risk assessment models, as these associations may differ and be obscured when only composite outcomes are used.

## Notes

Supplement 1. **eFigure 1.** Severe Acute Outcomes in Infants Born <32 Weeks’ Gestation by Apgar Score and UA-pH Removing Countries With Missingness of UA-pH >65% **eFigure 2.** Severe Acute Outcomes in Infants Born <32 Weeks’ Gestation by Apgar Score and UA-pH Removing Countries With Missingness of UA-pH >50% **eFigure 3.** Severe Acute Outcomes in Infants Born <32 Weeks’ Gestation by Apgar Score and UA-pH Excluding Risk Adjustment for Maternal Age and Parity **eFigure 4.** Severe Acute Outcomes in Infants Born <32 Weeks’ Gestation by Apgar Score and UA-pH Excluding Management Variables **eFigure 5.** Severe Acute Outcomes in Infants Born <32 Weeks’ Gestation by Apgar Score and UA-pH Including Risk Adjustment for Preterm Labor **eFigure 6.** Sensitivity Analysis of Severe Acute Outcomes in Infants Born <32 Weeks’ Gestation by Apgar Score and UA-pH Including Hospital as Random Effect **eFigure 7.** Sensitivity Analysis of Severe Acute Outcomes in Infants Born <32 Weeks’ Gestation by Apgar Score and UA-pH on Cases With an UA-pH <7.10 **eTable 1.** Availability of Data on 5-Minute Apgar Score and Umbilical Artery pH by Country **eTable 2.** Comparison of Cases With and Without Missing Data on the 5-Minute Apgar Score and Umbilical Artery pH **eTable 3.** Unadjusted and Adjusted Relative Risks With 95% Confidence Intervals Associated With the Exposure and the Composite Outcome of Death or Severe Mortality and 3 Individual Components Presented in Figure 2

Supplement 2.
Nonauthor Collaborators

Supplement 3.
Data Sharing Statement
