# Supplementary material for: Apgar Score Plus Umbilical Artery pH and Adverse Neonatal Outcomes in Very Preterm Infants
Source: JAMA Netw Open. 2026 Feb 6;9(2):e2557913. doi: 10.1001/jamanetworkopen.2025.57913 (PMC12881985; doi:10.1001/jamanetworkopen.2025.57913)
Supplement: Supplement 1. — eFigure 1. Severe Acute Outcomes in Infants Born <32 Weeks’ Gestation by Apgar Score and UA-pH Removing Countries With Missingness of UA-pH >65% eFigure 2. Severe Acute Outcomes in Infants Born <32 Weeks’ Gestation by Apgar Score and UA-pH Removing Countries With Missingness of UA-pH >50% eFigure 3. Severe Acute Outcomes in Infants Born <32 Weeks’ Gestation by Apgar Score and UA-pH Excluding Risk Adjustment for Maternal Age and Parity eFigure 4. Severe Acute Outcomes in Infants Born <32 Weeks’ Gestation by Apgar Score and UA-pH Excluding Management Variables eFigure 5. Severe Acute Outcomes in Infants Born <32 Weeks’ Gestation by Apgar Score and UA-pH Including Risk Adjustment for Preterm Labor eFigure 6. Sensitivity Analysis of Severe Acute Outcomes in Infants Born <32 Weeks’ Gestation by Apgar Score and UA-pH Including Hospital as Random Effect eFigure 7. Sensitivity Analysis of Severe Acute Outcomes in Infants Born <32 Weeks’ Gestation by Apgar Score and UA-pH on Cases With an UA-pH <7.10 eTable 1. Availability of Data on 5-Minute Apgar Score and Umbilical Artery pH by Country eTable 2. Comparison of Cases With and Without Missing Data on the 5-Minute Apgar Score and Umbilical Artery pH eTable 3. Unadjusted and Adjusted Relative Risks With 95% Confidence Intervals Associated With the Exposure and the Composite Outcome of Death or Severe Mortality and 3 Individual Components Presented in Figure 2 [file jamanetwopen-e2557913-s001.pdf]

Supplemental Online Content

Ehrhardt H, Behboodi S, Maier RF, et al; on behalf of the EPICE/SHIPS Research Group. Apgar score plus umbilical artery pH and adverse neonatal outcomes in very preterm infants. *JAMA Netw Open*. 2026;9(2):e2557913.
doi:10.1001/jamanetworkopen.2025.57913

eFigure 1. Severe Acute Outcomes in Infants Born <32 Weeks' Gestation by Apgar Score and UA-pH Removing Countries With Missingness of UA-pH >65%
eFigure 2. Severe Acute Outcomes in Infants Born <32 Weeks' Gestation by Apgar Score and UA-pH Removing Countries With Missingness of UA-pH >50%
eFigure 3. Severe Acute Outcomes in Infants Born <32 Weeks' Gestation by Apgar Score and UA-pH Excluding Risk Adjustment for Maternal Age and Parity
eFigure 4. Severe Acute Outcomes in Infants Born <32 Weeks' Gestation by Apgar Score and UA-pH Excluding Management Variables
eFigure 5. Severe Acute Outcomes in Infants Born <32 Weeks' Gestation by Apgar Score and UA-pH Including Risk Adjustment for Preterm Labor
eFigure 6. Sensitivity Analysis of Severe Acute Outcomes in Infants Born <32 Weeks' Gestation by Apgar Score and UA-pH Including Hospital as Random Effect
eFigure 7. Sensitivity Analysis of Severe Acute Outcomes in Infants Born <32 Weeks' Gestation by Apgar Score and UA-pH on Cases With an UA-pH <7.10
eTable 1. Availability of Data on 5-Minute Apgar Score and Umbilical Artery pH by Country
eTable 2. Comparison of Cases With and Without Missing Data on the 5-Minute Apgar Score and Umbilical Artery pH
eTable 3. Unadjusted and Adjusted Relative Risks With 95% Confidence Intervals Associated With the Exposure and the Composite Outcome of Death or Severe Mortality and 3 Individual Components Presented in Figure 2

This supplemental material has been provided by the authors to give readers additional information about their work.

© 2026 Ehrhardt H et al. *JAMA Network Open*

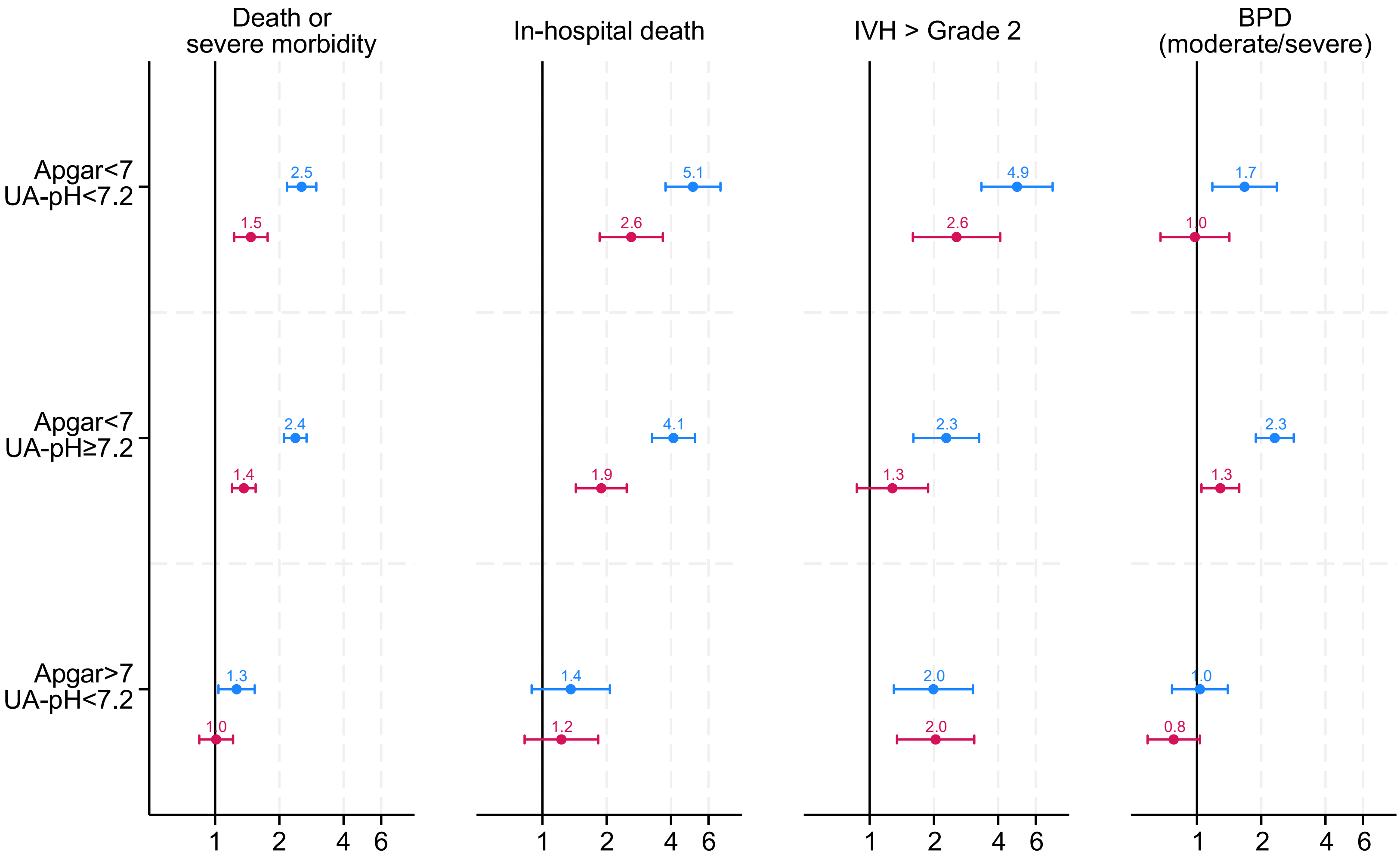

© 2026 Ehrhardt H et al. *JAMA Network Open*

eFigure 1

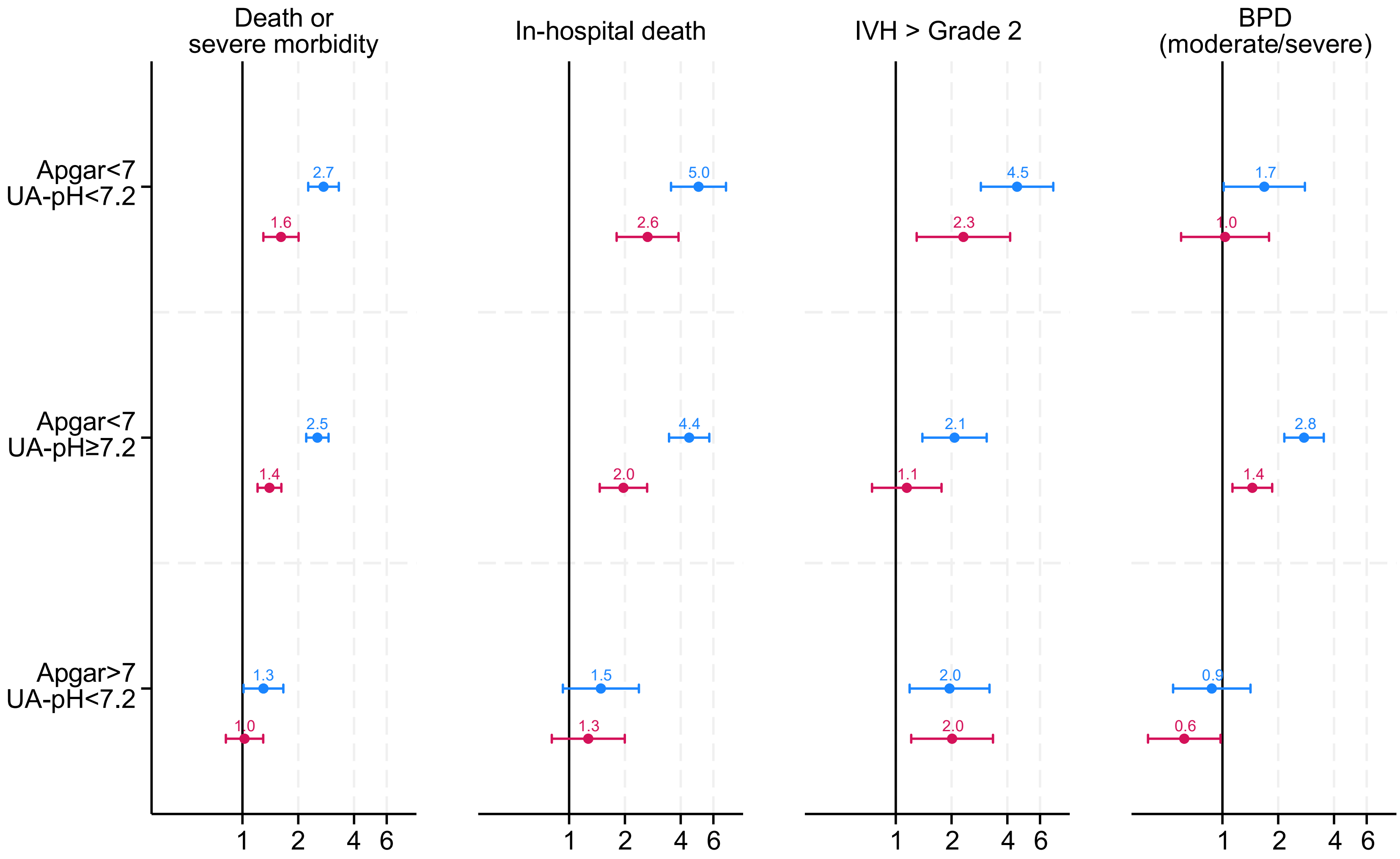

© 2026 Ehrhardt H et al. *JAMA Network Open*

eFigure 2

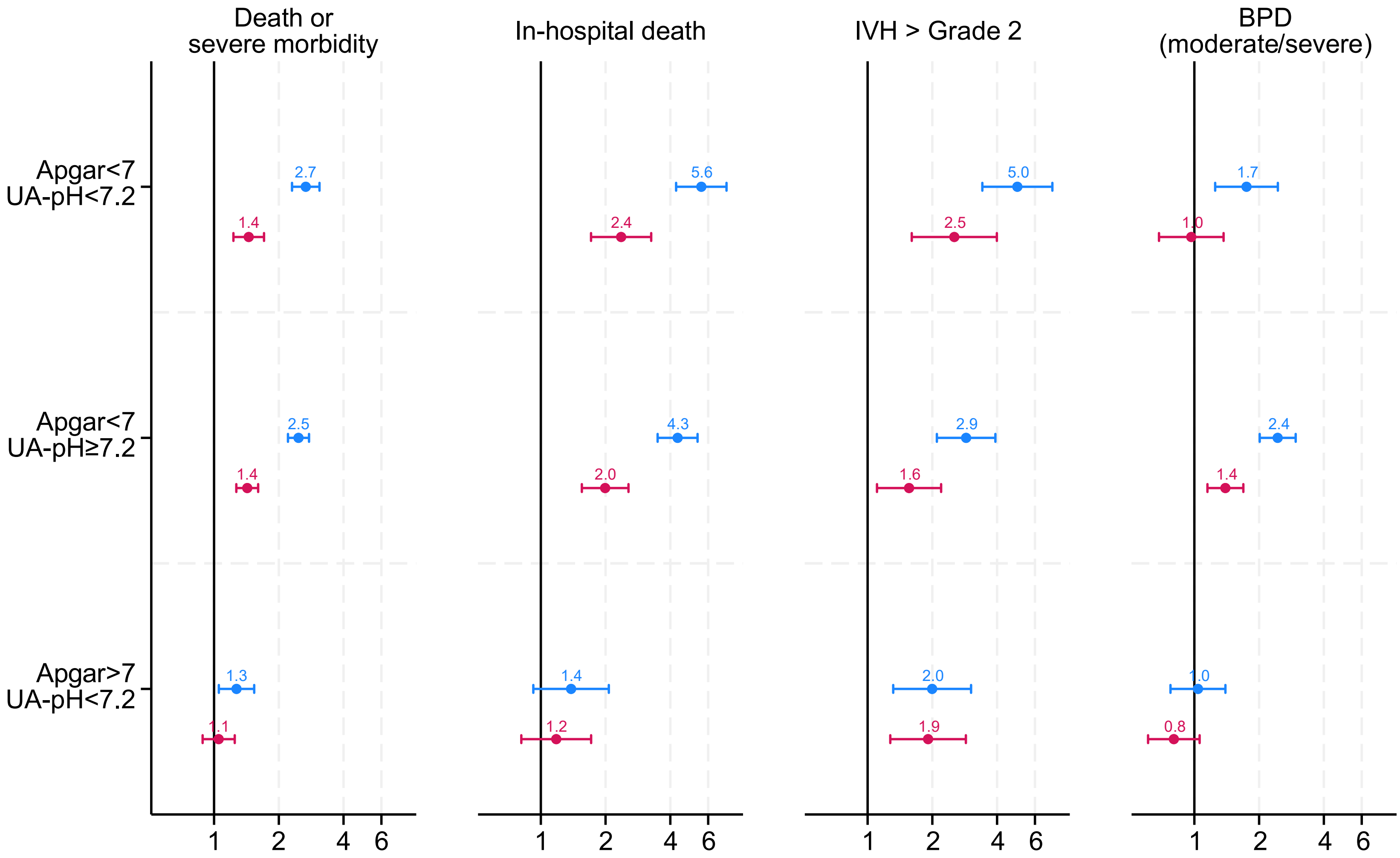

© 2026 Ehrhardt H et al. *JAMA Network Open*

eFigure 3

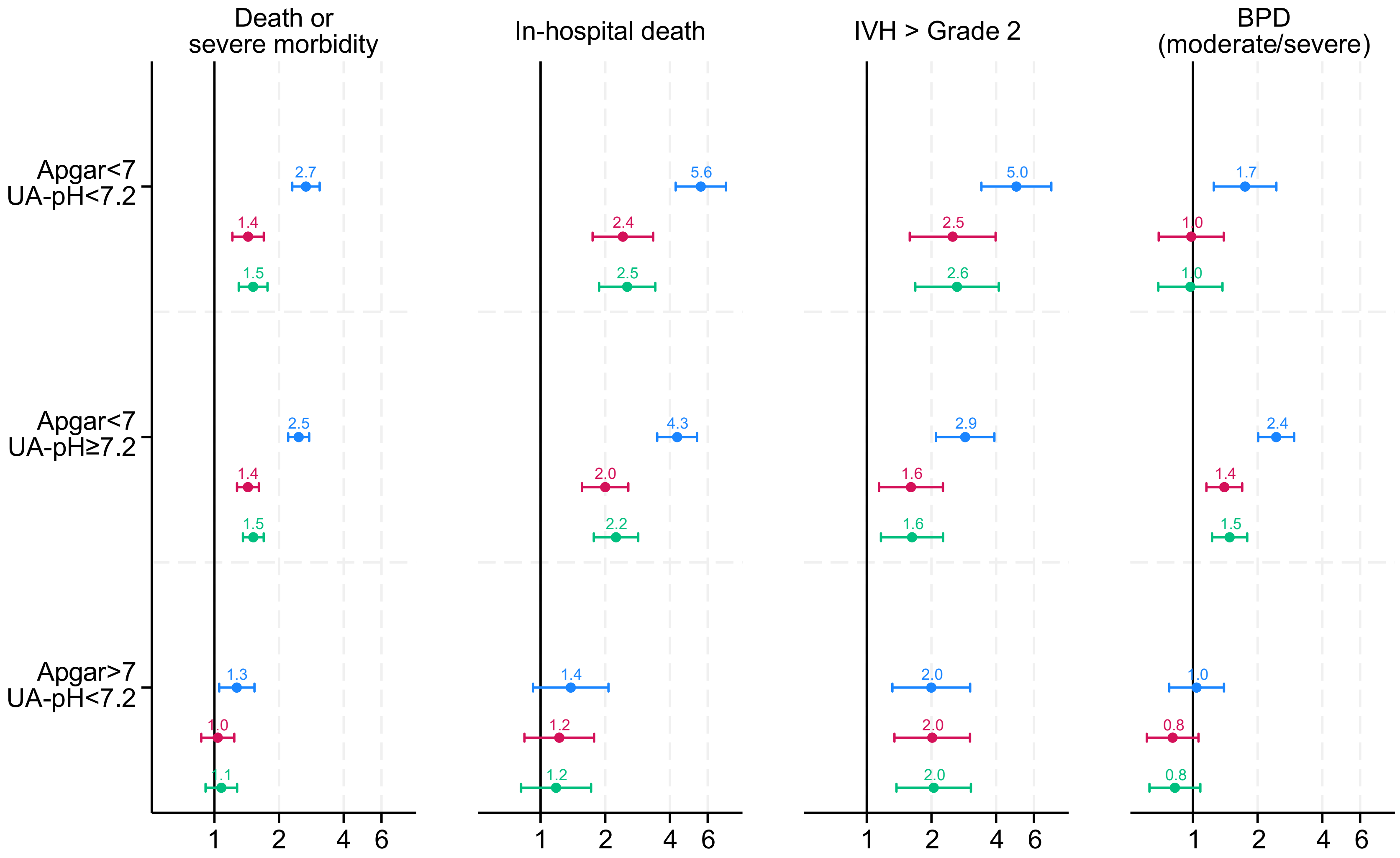

© 2026 Ehrhardt H et al. *JAMA Network Open*

eFigure 4

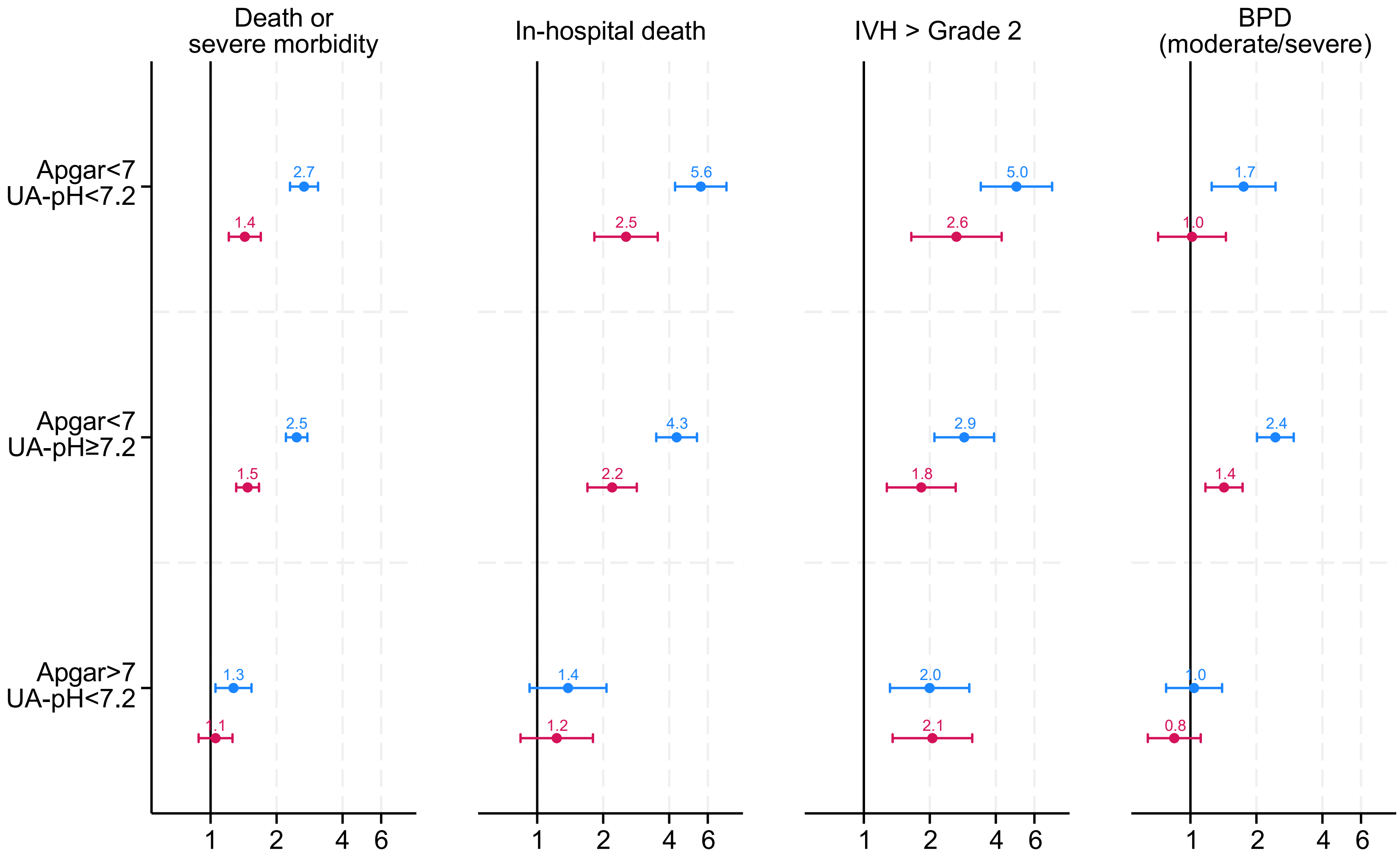

© 2026 Ehrhardt H et al. *JAMA Network Open*

eFigure 5

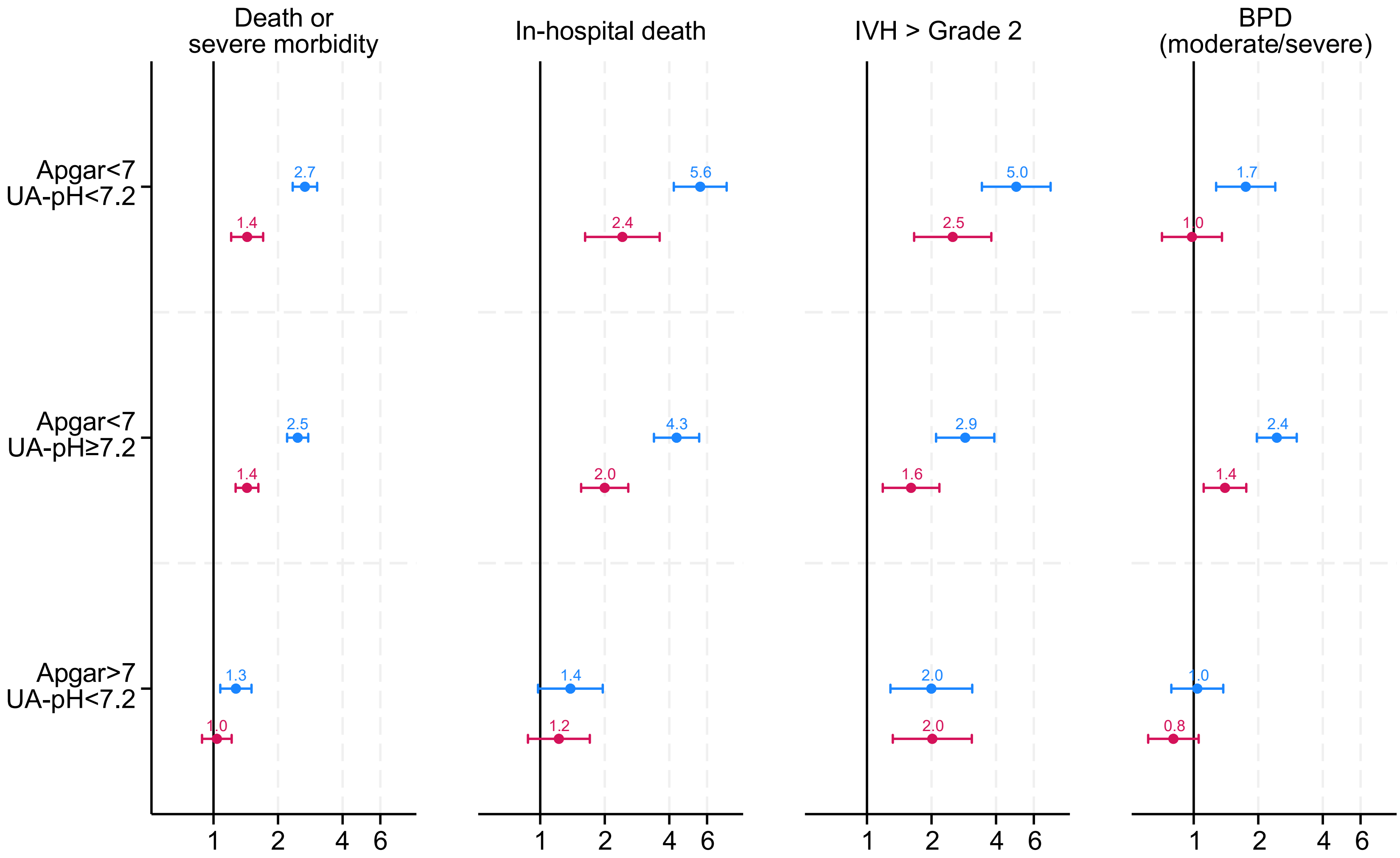

© 2026 Ehrhardt H et al. *JAMA Network Open*

eFigure 6

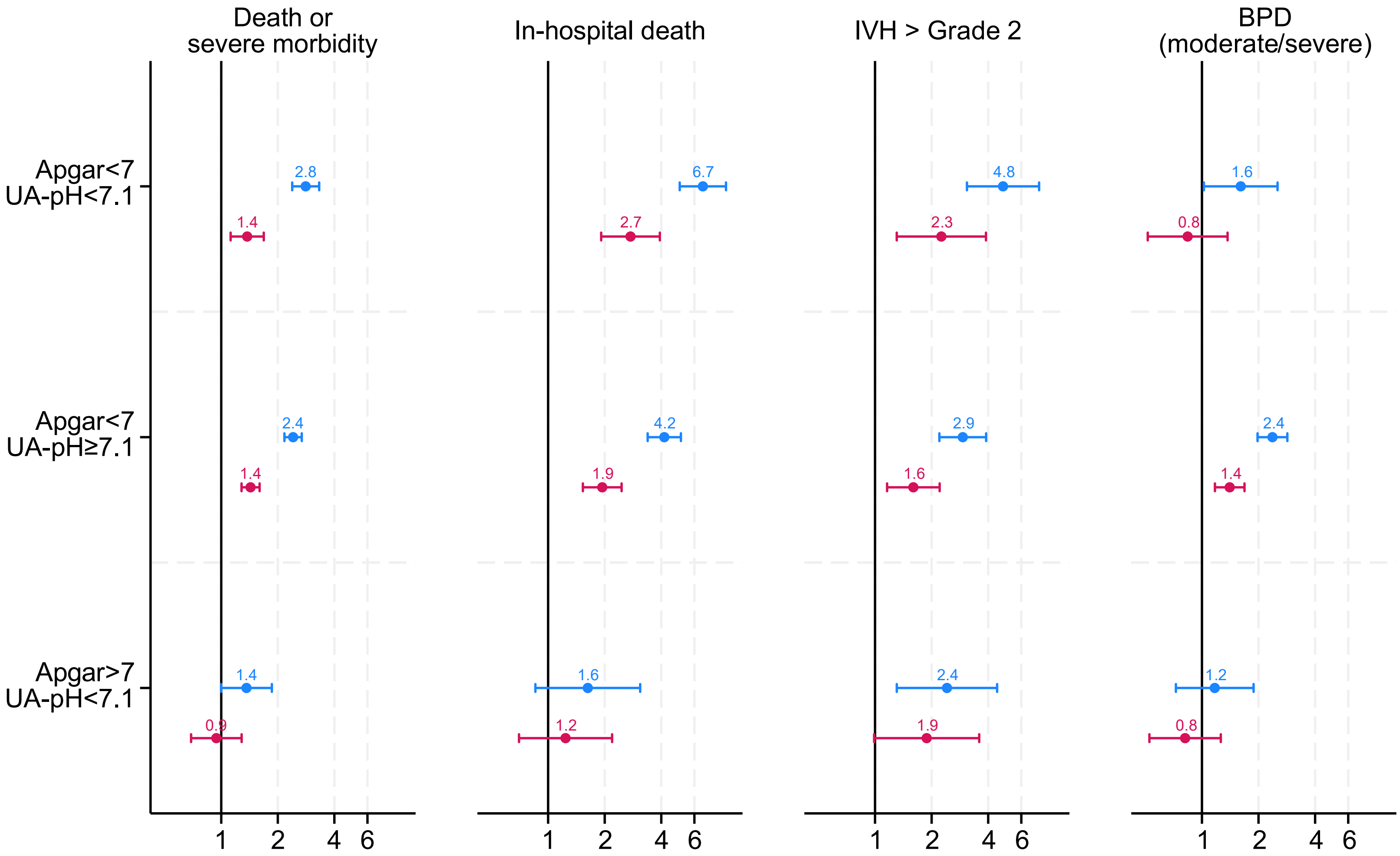

© 2026 Ehrhardt H et al. *JAMA Network Open*

eFigure 7

eFigure legends

eFigure 1. Severe acute outcomes in infants born <32 weeks´ gestation by Apgar score and UA-pH removing countries with missingness of UA-pH >70%. Sensitivity analysis of data from Figure 2 excluding Italy and Portugal for missingness of UA-pH >65%. Abbreviations: IVH, intraventricular hemorrhage; BPD, bronchopulmonary dysplasia; UA-pH, umbilical artery pH.

eFigure 2. Severe acute outcomes in infants born <32 weeks´ gestation by Apgar score and UA-pH removing countries with missingness of UA-pH >50%. Sensitivity analysis as in eFigure 1 excluding additionally Sweden and the United Kingdom for missingness of UA-pH >50%. Abbreviations: IVH, intraventricular hemorrhage; BPD, bronchopulmonary dysplasia; UA-pH, umbilical artery pH.

eFigure 3. Severe acute outcomes in infants born <32 weeks´ gestation by Apgar score and UA-pH excluding risk adjustment for maternal age and parity. Analyses from Figure 2 were executed excluding risk adjustment for maternal age and parity. Abbreviations: IVH, intraventricular hemorrhage; BPD, bronchopulmonary dysplasia; UA-pH, umbilical artery pH.

eFigure 4. Severe acute outcomes in infants born <32 weeks´ gestation by Apgar score and UA-pH excluding management variables. Analyses from Figure 2 were executed with risk adjustment for country only (displayed in blue), with additional adjustment for maternal age, perinatal variables (GA, SGA, sex, multiple birth, congenital anomaly), pregnancy complications (preterm rupture of membranes, hypertensive disorders of pregnancy), parity, mode of delivery, ANS and inborn status (red) and adjustments excluding management variables (green). Abbreviations: IVH, intraventricular hemorrhage; BPD, bronchopulmonary dysplasia; UA-pH, umbilical artery pH.

eFigure 5. Sensitivity analysis of severe acute outcomes in infants born <32 weeks´ gestation by Apgar score and UA-pH including hospital as random effect. Sensitivity analysis of data from Figure 2 including hospital as random effect. Abbreviations: IVH, intraventricular hemorrhage; BPD, bronchopulmonary dysplasia; UA-pH, umbilical artery pH.

eFigure 6. Sensitivity analysis of severe acute outcomes in infants born <32 weeks´ gestation by Apgar score and UA-pH including hospital as random effect. Sensitivity analysis of data from Figure 2 including hospital as random effect. Abbreviations: IVH, intraventricular hemorrhage; BPD, bronchopulmonary dysplasia; UA-pH, umbilical artery pH.

eFigure 7. Sensitivity analysis of severe acute outcomes in infants born <32 weeks´ gestation by Apgar score and UA-pH on cases with an UA-pH <7.10. Sensitivity analysis of data from Figure 2 with UA-pH cutoff of 7.10. Abbreviations: IVH, intraventricular hemorrhage; BPD, bronchopulmonary dysplasia; UA-pH, umbilical artery pH.

© 2026 Ehrhardt H et al. *JAMA Network Open*

eTable 1. Availability of data on 5-minute Apgar score and umbilical artery pH by country

	All live births	Data on Apgar & UA-pH	Missing data on Apgar and/or UA-pH	Missing data on Apgar	Missing data on UA-pH
Country	N=7,900	N=4,174	N=3,726	N=504	N= 3,535
Belgium	752	507 (67.4%)	245 (32.6%)	21 (2.79)	233 (30.98)
Denmark	351	209 (59.5%)	142 (40.5%)	13 (3.7)	141 (40.17)
Estonia	153	130 (85.0%)	23 (15.0%)	1 (0.65)	19 (12.42)
France	1,307	816 (62.4%)	491 (37.6%)	108 (8.26)	429 (32.82)
Germany	758	667 (88.0%)	91 (12.0%)	32 (4.22)	61 (8.05)
Italy	1,134	343 (30.2%)	791 (69.8%)	158 (13.93)	770 (67.9)
Netherlands	393	266 (67.7%)	127 (32.3%)	1 (0.25)	126 (32.06)
Poland	316	238 (75.3%)	78 (24.7%)	14 (4.43)	74 (23.42)
Portugal	724	89 (12.3%)	635 (87.7%)	6 (0.83)	624 (86.19)
United Kingdom	1,745	782 (44.8%)	963 (55.2%)	148 (8.48)	919 (52.66)
Sweden	267	127 (47.6%)	140 (52.4%)	2 (0.75)	139 (52.06)

Data are given as absolute numbers and percentages. Abbreviations: UA-pH, umbilical artery pH.

© 2026 Ehrhardt H et al. *JAMA Network Open*

eTable 2. Comparison of cases with and without missing data on the 5-minute Apgar score and umbilical artery pH

	Data on Apgar & UA-pH	Missing data on Apgar and/or UA-pH	Country-adjusted p-value	Unadjusted p-value
	N=4,174	N=3,726		
Maternal age (years)			0.633	<0.001
<25	678 (16.3%)	667 (18.0%)		
25-34	2,466 (59.3%)	1,984 (53.6%)		
≥35	1,018 (24.5%)	1,049 (28.4%)		
Maternal parity			0.142	0.60
Primiparous	2,335 (56.5%)	2,057 (55.9%)		
Multiparous	1,795 (43.5%)	1,620 (44.1%)		
Type of pregnancy			<0.001	0.007
Singleton	2,928 (70.2%)	2,509 (67.4%)		
Multiple	1,245 (29.8%)	1,216 (32.6%)		
Preeclampsia/eclampsia/HELLP			<0.001	<0.001
No	3,379 (82.6%)	3,194 (87.8%)		
Yes	711 (17.4%)	442 (12.2%)		
PPROM (>12h)			0.485	0.057
No	3,034 (74.1%)	2,756 (76.0%)		
Yes	1,060 (25.9%)	871 (24.0%)		
Any antenatal steroids			<0.001	<0.001
No	388 (9.4%)	667 (18.1%)		
Yes	3,747 (90.6%)	3,012 (81.9%)		
Inborn			<0.001	<0.001
No	389 (9.4%)	471 (13.5%)		
Yes	3,749 (90.6%)	3,012 (86.5%)		
Mode of delivery			<0.001	<0.001
Vaginal birth	1,160 (28.1%)	1,334 (36.4%)		
Vaginal instrumental	102 (2.5%)	83 (2.3%)		
Prelabour caesarean	1,774 (42.9%)	1,419 (38.7%)		
Intrapartum caesarean	1,098 (26.6%)	829 (22.6%)		
Sex of the infant			0.984	0.75
Male	2,249 (53.9%)	2,017 (54.2%)		
Female	1,925 (46.1%)	1,702 (45.8%)		
Gestational age (completed weeks)			<0.001	<0.001
<26	428 (10.3%)	737 (19.8%)		
26-27	655 (15.7%)	702 (18.8%)		
28-29	1,109 (26.6%)	870 (23.3%)		
30-31	1,982 (47.5%)	1,417 (38.0%)		
SGA (centiles, intrauterine charts)			0.713	0.64
<3	877 (21.0%)	756 (20.3%)		
3-9	480 (11.5%)	416 (11.2%)		
≥10	2,817 (67.5%)	2,546 (68.5%)		

© 2026 Ehrhardt H et al. *JAMA Network Open*

eTable 2 continued. Comparison of cases with and without missing data on the 5-minute Apgar score and umbilical artery pH

	Data on Apgar & UA-pH	Missing data on Apgar and/or UA-pH	Country-adjusted p-value	Unadjusted p-value
	N=4,174	N=3,726		
Severe congenital malformation			0.254	0.001
No	3,780 (90.6%)	3,451 (92.6%)		
Yes	394 (9.4%)	275 (7.4%)		
Outcomes				
Death or severe morbidity	1,068 (25.6%)	1,459 (39.2%)	<0.001	<0.001
In-hospital death	366 (8.8%)	740 (19.9%)	<0.001	<0.001
Death in the labour ward	54 (1.3%)	287 (7.7%)	<0.001	<0.001
Death after NICU admission	312 (7.6%)	453 (13.2%)	<0.001	<0.001
IVH >grade 2[1]	222 (5.5%)	279 (8.3%)	<0.001	<0.001
BPD (moderate/severe)[2]	493 (13.0%)	544 (18.4%)	<0.001	<0.001

Data are given as absolute numbers and percentages. Total number of patients differs for missing values for individual covariables due to death before diagnosis or missing data ([1]n=103 for IVH; [2]n=391 for BPD). p-values were calculated unadjusted and adjusted for country. Abbreviations: UA-pH, umbilical artery pH; HELLP, hemolysis, elevated liver enzymes, and low platelets; IVH, intraventricular hemorrhage; PPROM, preterm premature rupture of membranes; caesarean, caesarean section; SGA, small for gestational age; NICU, neonatal intensive care unit; IVH, intraventricular hemorrhage; BPD, bronchopulmonary dysplasia.

© 2026 Ehrhardt H et al. *JAMA Network Open*

eTable 3. Unadjusted and adjusted relative risks with 95% confidence intervals associated with the exposure and the composite outcome of death or severe mortality and 3 individual components presented in Figure 2

	**Death or severe morbidity**		**In-hospital death**		**IVH >grade 2**		**BPD (moderate/severe)**	
	RR (95%CI)	aRR (95%CI)	RR (95%CI)	aRR (95%CI)	RR (95%CI)	aRR (95%CI)	RR (95%CI)	aRR (95%CI)
Apgar≥7 & UA-pH ≥7.20	REF	REF	REF	REF	REF	REF	REF	REF
Apgar <7 & UA-pH <7.20	2.7 (2.3,3.1)	1.4 (1.2,1.7)	5.6 (4.3,7.3)	2.4 (1.7,3.3)	5.0 (3.4,7.2)	2.5 (1.6,4.0)	1.7 (1.2,2.4)	1.0 (0.7,1.4)
Apgar <7 & UA-pH ≥7.20	2.5 (2.2,2.8)	1.4 (1.3,1.6)	4.3 (3.5,5.4)	2.0 (1.6,2.6)	2.9 (2.1,3.9)	1.6 (1.1,2.3)	2.4 (2.0,3.0)	1.4 (1.2,1.7)
Apgar≥7 & UA-pH <7.20	1.3 (1.1,1.5)	1.0 (0.9,1.2)	1.4 (0.9,2.1)	1.2 (0.8,1.8)	2.0 (1.3,3.0)	2.0 (1.3,3.0)	1.0 (0.8,1.4)	0.8 (0.6,1.1)

Data from Figure 2 presented in tabulated form. Abbreviations: UA-pH, umbilical artery pH; age; IVH, intraventricular hemorrhage; BPD, bronchopulmonary dysplasia.

© 2026 Ehrhardt H et al. *JAMA Network Open*
