# Supplementary material for: Apgar Score Plus Umbilical Artery pH and Adverse Neonatal Outcomes in Very Preterm Infants
Source: JAMA Netw Open. 2026 Feb 6;9(2):e2557913. doi: 10.1001/jamanetworkopen.2025.57913 (PMC12881985; doi:10.1001/jamanetworkopen.2025.57913)
Supplement: Supplement 3. — Data Sharing Statement [file jamanetwopen-e2557913-s003.pdf]

Data Sharing Statement

Data
Data available: No

Additional Information
Explanation for why data not available: Access to the data of the EPICE and SHIPS cohort is currently not possible for researchers who are not members of the consortium, but EPICE is part of a H2020 project (RECAP, https://recap-preterm.eu/) to develop a platform for data sharing. The corresponding author is available for more information.
